# Design and psychometric evaluation of the collaborative coping with infertility questionnaire in candidate of assisted reproductive techniques

**DOI:** 10.1038/s41598-024-61607-0

**Published:** 2024-05-11

**Authors:** Marzie Reisi, Ashraf Kazemi

**Affiliations:** 1 School of Nursing and Midwifery, Shahrekord University of Medical Sciences, Shahrekord, Iran; 2 Nursing and Midwifery Care Research Center, Reproductive Health Department, School of Nursing and Midwifery, Isfahan University of Medical Sciences, Isfahan, Iran

**Keywords:** Collaborative, Coping, Infertility, Psychometric, Reproductive techniques, Assisted, Psychology, Health care

## Abstract

Evaluating couples’ coping with infertility and its impact on their mental health is valuable in designing supportive programs. Since infertility is a shared problem in married life, coping with it requires collaborative coping strategies. Therefore, the aim of the present study was to design and psychometrically evaluate the collaborative coping with infertility questionnaire (CCIQ) in candidates of assisted reproductive techniques (ART). The exploratory factor analysis of a 27-item questionnaire designed based on the Likert scale in the Persian language was evaluated through the principal component analysis method in a cross-sectional study conducted on 200 couples who volunteered for ART. The cut-off point of factor loadings was considered 0.4. Furthermore, the criterion validity of the questionnaire was evaluated using a 12-item revised Fertility Adjustment Scale (R-FAS) and its relationship with the score of the CCIQ. Moreover, the internal consistency of the questionnaire was evaluated using Cronbach’s alpha correlation coefficient. In the exploratory factor analysis, 20 items with a factor loading above 0.4 were extracted under three factors. The three extracted factors with a value above one explained 43.78% of the variance of CCIQ. The factor loading of the accepted items ranged between 0.402 and 0.691. External reliability was confirmed with Cronbach’s alpha coefficient of 0.98. The relationship between CCIQ and R-FAS score was significant (p < 0.0001). The results of the study showed that the 20-item CCIQ enjoyed acceptable validity and reliability in the three dimensions of ‘dynamic interaction,’ ‘reorganizing married life goals,’ and ‘perception about infertility,’ which can be used to evaluate collaborative coping with infertility questionnaire in ART candidates.

## Introduction

Infertility, with a prevalence of approximately 15%^1^, imposes a significant psychosocial burden on the couple involved. Social isolation and the stigma of infertility in couples with infertility^2^ are associated with increased levels of stress, anxiety, and depression^3,4^, which reduce the quality of married life^5^. In addition, the increased likelihood of domestic violence^6^ and decreased quality of sexual relations^7,8^ threaten the durability of married life in these couples^9^. Therefore, infertility is considered a crisis in the life of the couple involved and needs to be managed.

The extensive effects of infertility on different aspects of life oblige the involved couples to consider it a priority and find a solution for it in their married life. However, the negative impacts of infertility on various dimensions of married life, along with the tension of the treatment, increase the need to adopt adaptive coping strategies according to the conditions.

Far-reaching and efficacious efforts have been made to address the infertility problem. Many involved couples experience a successful pregnancy using medical methods and corrective surgeries. However, approximately 30–35% need to use assisted reproductive technologies (ART) following the failure of these treatments.

In this regard, ART has increased the hope of solving the infertility problem; however, the uncertainty of the treatment result for each treatment cycle and the high cost of these technologies increases the psychological burden of infertility^4,10^ and the need for efficacious adaptive strategies.

Depending on their social and cultural background^11^, couples employ myriad strategies to deal with infertility crises, which can have various short-term and long-term effects on their mental health^12,13^. Identifying these strategies can be advantageous for formulating counseling programs.

Since infertility is a couple-based problem, each partner’s psychological conditions and coping strategies might affect the other partner’s coping and mental health^14^.

A study has shown that in couples who are candidates for ART, a better quality of life in women is predicted with their partner’s more capabilities of identifying and describing emotions. It is also reported in this study that the better quality of life of each couple is related to the quality of life of the other^15^.

These reports suggest that interactive models are more appropriate than individual models when explaining and evaluating couples’ coping strategies toward infertility. When explaining the stress management process, the Systemic-Transactional Model emphasizes the impact of one partner’s conditions on the behavior of the other as well as the dyadic coping^16^.

It is more important to use these models to cope with couples who are candidates for ART and need mutual support more than ever before. Because these couples experience more psychological emotions than before entering these treatments^17^. The evaluation of their couple coping can be used in formulating counseling programs for ART candidates.

Various tools have been developed and applied to measure coping in couples with infertility some of which have been compiled merely to measure women's coping, such as the Coping Scale for Infertile Women^18^ and Coping Scale for Infertility-Women^19^. Another valid tool that is used to assess coping in both genders is the revised version of COPE^20^, which was validated for use in different countries and used in numerous researches. In compiling these tools, a couple-based approach is not taken into account to evaluate coping with infertility.

Previous efforts to develop and validate the dyadic coping assessment tool included the Dyadic Coping Questionnaire^21^ and Dyadic Coping Inventory^22^. The application of translated versions of these tools has been confirmed in different populations^23–26^.

The Dyadic Coping Questionnaire, with 41 items, measures the dyadic coping method, including dyadic coping behavior: supporting, delegated, negative, and common behavior^21^. Another questionnaire, the Dyadic Coping Inventory, focuses on couples’ communication and their perception of support from the spouse^22^.

These questionnaires have been designed based on the systemic-transactional model; however, they are not focused specifically on couples with infertility and the psychosocial burden of ART candidate couples. These Couples are exposed to major social problems, which can challenge their gender identity; consequently, they need dissimilar coping methods whose measurement is essential for formulating counseling programs for adjustment to infertility. Therefore, the present study aimed to design and develop a collaborative coping with infertility questionnaire (CCIQ) in couples who are candidates for ART.

## Materials and methods

This study was conducted in two phases with the approval of the Ethics Committee of Isfahan University of Medical Sciences (IR.MUI.NUREMA.REC.1400.019) from June 2022 to July 2023 in Isfahan, Iran. The participants in this study were couples with infertility referred to the Fertility and Infertility Center to receive assisted reproductive techniques (ART), and they had not yet entered the ovulation stimulation phase.

The inclusion criteria included not being illiterate, having no third partner in treatment (such as gamete donation or surrogate mother), having no major psychiatric diseases such as schizophrenia and bipolar psychosis (based on the medical records in the file), the participation of both partners (man and woman), and having no children.

*The First phase*: Development of the CCIQ.

In order to compile the questionnaire items, a study was conducted using the qualitative content analysis approach and interviews with 18 couples with infertility (36 individuals) at the Isfahan Infertility and Fertility Center. Based on the results of that study, an initial 63-item questionnaire was designed on a five-point Likert scale (0–4), including 4: always, 3: often, 2: sometimes, 1: rarely, and 0 = never, in Persian. A higher score indicated more use of collaborative coping.

The tool’s face and content validity were evaluated through qualitative and quantitative methods using the opinions of 14 experts in the fields of psychometrics (2 individuals), psychology (3 individuals), reproductive health (2 individuals), psychiatric nursing (2 individuals), social psychology (2 individuals), health psychology (2 individuals), and psychiatry (1 individual).

The questionnaire’s qualitative face validity was evaluated and confirmed by applying experts’ opinions regarding item wording, easy understanding, grammar, ease of completion, and logical sequence of items. Moreover, the questionnaire was provided to 6 couples with infertility, and its face validity was evaluated in terms of clarity and ease of completion.

In order to evaluate the quantitative face validity, the Impact Item Index was calculated. To this end, a checklist on a 5-point Likert scale (1–5) ranging from 1: not important at all to 5: very important was used. The impact factor of each item was determined by calculating the relative importance and frequency.

To evaluate the content validity of the questionnaire through a qualitative method, experts’ opinions regarding the concept and coverage of the subject dimensions were collected, and 7 items were removed after applying their opinions.

To evaluate the content validity ratio (CVR), the experts were requested to choose one of 3 answers for each item, including necessary (3 scores), useful but not necessary (2 scores), and not necessary (1 score). The calculated formula of CVR was (Ne-N/2)/(N/2), where Ne represents the number of experts who evaluated the essential items, and N denotes the number of experts. Afterward, based on the formula of Lawshe’s suggestion, the items with CVR higher than 0.51 were maintained, and 2 items were removed.

To evaluate the content validity index (CVI), the Waltz and Bausell method was used, and experts’ opinions on the relevance, clarity, and simplicity of each item were collected using a checklist on a 4-point Likert scale (1–4). The CVI was calculated by dividing the number of experts who rated the items as 3 or 4 by the total number of experts; CVI = (sum of items rated 3 or 4) / (the number of all the responses).

The minimum acceptable score for CVI was 0.79. The items with scores between 0.79 and 0.70 were modified, and those with scores lower than 0.70 were removed. a questionnaire with 27 items was obtained. The mean CVI for the questionnaire and all its items were 0.91 and 0.84, respectively.

To determine the tool’s reliability, the questionnaire was completed in a pilot study by 20 couples with infertility and repeated in 3 weeks to determine its repeatability. To evaluate internal consistency, Cronbach’s alpha coefficient was calculated, and the questionnaire was confirmed with 27 items with a coefficient of 0.81. Moreover, a two-way random method with a confidence interval of 0.95 was used to determine the external reliability of the ICC coefficient. The repeatability of the tool was also confirmed with a coefficient of 0.833. This ratio was 0.801 and 0.840 for men and women, respectively.

*The second phase:* Construct validity of the CCIQ.

The construct validity of the 27-item questionnaire was evaluated through a cross-sectional study on couples undergoing ART. The sample size was determined based on the ratio of at least 7 couples per item^27^. Consequently, 200 Iranian couples (400 individuals) undergoing ART were included in the study using the convenience sampling method.

All eligible couples were invited to participate in the research, and after obtaining informed consent, background information was completed. The designed questionnaire and a revised Fertility Adjustment Scale (12-item R-FAS) were completed as a self-report by couples to evaluate adjustment to infertility. The R-FAS has been designed with 12 items on a 6-point Likert scale from totally disagree (1) to Totally agree (6). A higher score indicates less adjustment^28^. Convergent and concurrent criterion validity was assessed by evaluating the relationship between couples’ adjustment to infertility and their collaborative coping.

### Data analysis

Data were analyzed using SPSS software version 19. Exploratory factor analysis was used to assess the construct validity. The adequacy of the sample size and the correlation between the extracted factors were investigated using the Kaiser–Meyer–Olkin and Bartlett’s tests. The exploratory factor analysis was performed using the principal component analysis with Varimax rotation. Moreover, the scree plot was used to determine the number of extracted factors.

The cut-off point of factor loadings was considered 0.4.

Considering the items with an acceptable factor loading in more than one factor, the conceptual similarity was considered to sort the items in one factor. Afterward, factors were labeled based on the concepts of the items and considering the labels of qualitative subcategories and categories, including ‘dynamic interaction’, ‘reorganizing married life goals,’ and ‘perception of infertility’. After assessing construct validity, Cronbach’s alpha coefficient of the entire questionnaire and its factors was recalculated.

### Ethics approval and consent to participate

All procedures performed on participants were in accordance with the ethical standards of the Isfahan University of Medical Sciences, and informed consent was obtained from all participants**.**

## Results

Of the 223 couples invited to participate in the study, 200 eligible couples accepted the invitation. The background characteristics of these couples (400 individuals) are presented in Table 1.

**Table 1:** The baseline characteristics of the participants (N = 400).

	Mean (SD) or number (%)
Age (mean)	
Women	34.66 (5.4)
Men	37.6 (5.9)
Education level in women (%)	
High school or lower	23 (11.5)
Diploma	82 (41.0)
Bachelor's degree and higher	95 (47.7)
Education level in men (%)	
High school or lower	41 (20.5)
Diploma	92 (46.0)
Bachelor's degree and higher	67 (33.5)
Employed (%)	
Women	90 (45.0)
Men	186 (93.0)
Cause of infertility (%)	
Female infertility	99 (48.8)
Male infertility	78 (38.4)
Unexplained	26 (12.8)
Duration of infertility (years)	7.9 (4.7)
Fertility adjustment (mean)	
Women	36.9 (1.0)
Men	40.1 (.65)

The results of exploratory factor analysis using the Kaiser-Meyer-Elkin test (0.939) and Bartlett’s sphericity test (P < 0.0001) showed that the sample size was suitable for factor analysis. Moreover, based on the Kaiser criterion, three factors with a value above one were determined, which explained a total of 49.46% of the variance (Table 2). Evaluation of the scree diagram showed that three factors could be extracted. The items’ factor loading was in the range of 0.431–0.813.

**Table 2:** Varimax rotation on the factors.

Total variance explained									
Component	Initial eigenvalues			Extraction sums of squared loadings			Rotation sums of squared loadings		
	Total	% of Variance	Cumulative %	Total	% of Variance	Cumulative %	Total	% of Variance	Cumulative %
1	13.52	37.56	37.56	13.52	37.56	37.56	12.66	35.17	35.17
2	2.24	6.23	43.79	2.24	6.23	43.79	2.70	7.51	42.68
3	2.04	5.67	49.46	2.04	5.67	49.46	2.44	6.78	49.46

The first factor included 8 items with factor loading between 0.525 and 0.668, the second included 7 items with factor loading between 0.402 and 0.691, and the third included 5 items with factor loading between 0.485 and 0. 616 (Table 3). Seven items were removed due to their factor loading of less than 0.4. Cronbach's alpha coefficient value was 0.98 for the total scale, 0.810 for factor 1, 0.792 for factor 2, and 0.747 for factor 3.

**Table 3:** Loading of the items.

Items		Load factors		
		Factor 1	Factor 2	Factor 3
1	My spouse and I talk about our dreams about the future child	**0.587**	0.329	0.346
2	I understand my spouse’s need to love other’s children	**0.625**	0.020	− 0.105
3	If talking about infertility upsets us, we postpone it	**0.648**	0.146	− 0.019
4	We try not to let other’s judgments influence our relationships	**0.569**	0.067	0.101
5	We seek joint activities (such as sports and entertainment) to find peace	0.401	**0.587**	− 0.035
6	For me and my spouse, it doesn’t matter which of us the infertility problem is related to	− 0.216	− 0.003	**0.601**
7	We plan pleasure programs to maintain our married life	0.446	**0.663**	0.019
8	We plan to perform new activities in our married life	0.063	**0.691**	0.117
9	We provide each other with the conditions to achieve our goals	0.555	**0.622**	0.046
10	We support each other to create happiness for each other	**0.530**	0.083	− 0.057
11	We allocate time for our personal interests	0.120	**0.527**	0.055
12	We try to find a suitable solution to reduce each other’s worries	**0.638**	0.058	0.047
13	We have strengthened our relationships with happy infertile couples	0.307	**0.402**	0.040
14	We try to think that we are a different couple for having children, not an imperfect couple	0.054	0.230	**0.506**
15	We give each other time to accept infertility	0.015	0.171	**0.544**
16	We ensure each other of being together to solve infertility problems	0.341	0.200	0.058
17	Along with trying to get treatment, we plan for other life issues	0.154	**0.551**	0.110
18	We plan to solve the infertility problem and treat it	0.304	0.049	0.300
19	We try to trust each other to solve the infertility challenges	**0.668**	0.130	0.186
20	We consider infertility a problem that can be solved	0.386	0.058	**0.616**
21	Due to infertility, I have given up my opportunities for strengthening other aspects of life (reverse)	− 0.536	− 0.094	− 0.224
22	We allocate time to listen to each other’s concerns about infertility	**0.639**	0.225	0.118
23	We are concerned about other’s judgments about infertility. (R)	0.090	0.190	**0.485**
24	We accept the possibility of infertility treatment failure	0.309	0.110	− 0.578
25	My spouse and I try to make our shared dreams come true	0.307	0.229	− 0.134
26	We avoid topics that increase our concerns about infertility	0.309	0.210	0.066
27	My spouse and I prefer to spend time together than with others	0.340	0.309	0.189

Note: 7 items have been unaccepted (items 16, 18, 21, 24, 25, 26, 27).

Factor 1: Dynamic interaction; factor 2: Reorganizing married life goals; Factor 3: perception about infertility.

Significant values are given in bold.

The three extracted factors were labeled ‘dynamic interaction’, ‘reorganizing married life goals’, and ‘perception of infertility’. Investigating the relationship between CCIQ score and fertility adaptation showed that the R-FAS score was related to CCIQ score (ß = − 0.71, p < 0.0001, CI − 0.61 to − 0.74) by adjusting the results with age, education level, and duration and the cause of infertility. Moreover, the relationship between the R-FAS score with the dimensions of the dynamic interaction, reorganizing married life goals, and perception of infertility (p < 0.0001) was inverse and significant (Table 4).

**Table 4:** Relationship between collaborative coping with infertility and fertility adjustment.

	Fertility adjustment			
	Beta	CI 95%		Sig
Age	0.08	− 0.05	0.09	ns
Education level	0.04	− 1.10	3.06	ns
Duration of infertility	0.09	− 0.04	1.1	ns
Male factor infertility	0.16	0.12	9.26	0.02
Female factor infertility	0.12	− 0.55	2.73	ns
Unexplained infertility	0.10	− 0.09	1.50	ns
Cooperative coping with infertility	− 0.71	− 0.61	− 0.74	< 0.0001
Dynamic interaction	− 0.54	− 0.44	− 0.74	< 0.0001
Perception about infertility	− 0.85	− 0.91	− 1.94	< 0.0001
Reorganizing married life goals	− 0.38	− 0.61	− 0.19	< 0.0001

## Discussion

This study aimed to design and psychometrically evaluate the CCIQ in ART candidates. The results showed that the questionnaire with 20 items in the three dimensions of dynamic interaction, reorganizing married life goals, and perception of infertility had acceptable validity and reliability.

Dynamic interaction was one of the dimensions related to collaborative coping with infertility. Strengthening positive dynamic interaction as mutual understanding and perceiving of needs through enriching the interpersonal relationships between couples with infertility increases the sense of belonging^29^. Understanding the feelings and social support from the spouse improves marital quality^30^ and may reduce feelings of emptiness and unfulfilled life in couples with infertility.

A study has shown that the feelings of women and men about infertility vary. In many, particularly traditional, societies, infertility is mainly attributed to women, and they experience psychosocial burdens caused by infertility, such as social pressure, stigma, and rejection more than men^31^. While from the men’s point of view, infertility challenges their masculinity^32^. It has also been indicated that most couples lack an accurate understanding of their partner’s feelings and psychological needs, which deprives them of the opportunity to share their feelings and understand each other’s needs^33^. It also leads to their failure to gain the necessary insight to provide emotional needs and psychological support to each other since, in order to create an effective and positive relationship, it is essential for both parties to identify each other’s needs and adopt appropriate behavior according to the psychological and emotional conditions of their partner.

In addition, the observed relationship between the couple's dynamic interaction with coping with infertility confirms that positive interaction between couples can reduce the infertility crisis in married life. Consequently, assessing the dynamic interaction in the ART candidate couple reveals the couple’s need to adopt a mutual and positive relationship.

Another dimension extracted from collaborative coping with infertility was reorganizing married life goals. Fertility is a mutual and central goal in married life, and failure in it may damage couples’ emotional relationships. Therefore, planning to achieve common goals in married life may bridge this gap and improve emotional relationships between partners.

Although in this study the relationship between applying collaborative coping with infertility and the quality of the couple’s relationship has not been evaluated, the relationship between reorganizing married life goals and fertility adjustment shows that this strategy may improve relationships between couples through greater adaption to infertility.

Infertility is considered an obstacle to routine life for couples who intend to have children; as a result, the feeling of emptiness and futility in married life has been one of the recurring experiences among couples with infertility in different societies^34,35^. Accordingly, planning to deal with dimensions of life, which can be associated with greater health and well-being in married life, can be an efficient strategy to maintain and improve physical and mental health.

Measuring coping with infertility through goal replacement has been considered in some questionnaires^20^. This study showed that coping assessment through establishing common goals is useful in couples dealing with infertility.

Goal substitution has been an adaptive coping strategy among some men and women with infertility issues. However, formulating common goals in child-free situations may enhance this effect. It is believed that the couple’s common goals and their efforts to solve the shared problem result in a dynamic married life^36^. In the psychometrics of the dynamic coping inventory, which was developed to evaluate the understanding of couple relationships, the items related to joint efforts to solve problems had an acceptable factor load^37^.

The extraction of the perception of infertility dimension shows that couples need to create a shared rational approach toward infertility in order to cope with it. Despite the stereotypical perception of infertility in some societies, couples suffer high social pressures^38^. In many societies, fertility is defined as an imperative goal for married life, and infertility is considered an unfulfilled life^34^. This view of infertility exposes couples to a more severe crisis when faced with infertility and jeopardizes their mental health more than others^39^. There is a belief that a logical and realistic perception of a problem paves the way for solving it and is associated with greater adjustment to conditions causing crisis^40^.

The correlation of adjustment to infertility with the dimension of attitude towards infertility shows the applicability of CCIQ in predicting adaptive coping and mental health of couples. Moreover, this finding confirms that couples with infertility can better adapt to it as long as they have a reasonable and non-exaggerated perception of infertility; a view based on which couples with infertility believe that they enjoy a customary yet different life. A study showed that the belief of couples with infertility in enjoying a normal but different life was associated with a higher quality of life and better psychological adjustment. The present study reinforces the normalization view as an effective strategy for coping with infertility^39^, which can be considered as organizing a rational perception of infertility in couples.

The results of this study showed that the developed CCIQ can be used to evaluate collaborative coping with infertility in ART-candidate couples, taking into account their special psychosocial challenges. However, the limitations of this study restrain using this questionnaire in other populations.

The first limitation is conducting the study in a society where couples with infertility lived isolated more than other couples due to social stigma that limited their social relationships. Moreover, this study was conducted on couples who referred to the study setting to receive infertility treatment. Therefore, these results cannot be generalized to couples with infertility who have no hope of infertility treatment or have not taken any treatment. Another limitation of the study was the absence of a test for the divergent and convergent validity of the CCIQ.

In conclusion, this study showed that collaborative coping strategies could be evaluated in three areas: dynamic interactions, reorganizing married life goals, and perception of infertility. The developed valid and reliable 20-item questionnaire can be utilized to measure coping through the collaborative approach in couples who were candidates for ART.

## Data Availability

Data and material are available on request from the corresponding author.
